# *Siegesbeckia pubescens* Makino inhibits Pam_3_CSK_4_-induced inflammation in RAW 264.7 macrophages through suppressing TLR1/TLR2-mediated NF-κB activation

**DOI:** 10.1186/s13020-018-0193-x

**Published:** 2018-07-05

**Authors:** Wei Sang, Zhangfeng Zhong, Kegang Linghu, Wei Xiong, Anfernee Kai Wing Tse, Wai San Cheang, Hua Yu, Yitao Wang

**Affiliations:** 1 Institute of Chinese Medical Sciences, State Key Laboratory of Quality Research in Chinese Medicine, University of Macau, Macao, China; 2 HKBU Shenzhen Research Center, Shenzhen, Guangdong China; 3 School of Chinese Medicine, Hong Kong Baptist University, Kowloon Tong, Hong Kong China; 4 Guangdong Key Laboratory for Research and Development of Natural Drugs, Guangdong Medical University, Zhanjiang, China; 5 Academy for Advanced Interdisciplinary Studies, Southern University of Science and Technology, Shenzhen, Guangdong China; 6 Institute of Chinese Medical Sciences, University of Macau, Room 8008, Building N22, Avenida da Universidade, Taipa, Macao SAR China; 7 Institute of Chinese Medical Sciences, University of Macau, Room 1050, Building N22, Avenida da Universidade, Taipa, Macao SAR China

**Keywords:** *Siegesbeckia pubescens* Makino, Pam_3_CSK_4_, Inflammation, Toll-like receptor 1/2, NF-κB

## Abstract

**Background:**

*Siegesbeckia pubescens* Makino (SP) is one of the important plant origins for the anti-inflammatory Chinese herbal medicine of Siegesbeckiae Herba. The current investigations indicated that the anti-inflammatory effects of SP were associated with the toll-like receptors (TLRs)-mediated nuclear factor-κB (NF-κB) and the mitogen-activated protein kinase (MAPK) signaling pathways.

**Methods:**

Raw 264.7 macrophages were pretreated with the 50% ethanol extract of SP (SPE, 50–200 µg/mL) and then co-treated with Pam_3_CSK_4_ (200 ng/mL) for another 12 h. The inhibitory effect of SPE on Pam_3_CSK_4_-stimulated NO release and post-inflammatory cytokines secretions were determined using Griess reagent and Elisa kits, respectively. The influence of SPE on NF-κB and MAPKs signaling relevant proteins was measured by Western blotting analysis, while the intracellular nitric oxide (NO) generation and NF-κB/p65 nuclear translocation were determined using Leica TCS SP8 laser scanning confocal microscope. Moreover, the effect of SPE on luciferase reporter gene in NF-κB-luc DNA transfected raw 264.7 cells was determined using the Dual-Glo luciferase assay system kit.

**Results:**

SPE dose-dependently (50–200 µg/mL) attenuated Pam_3_CSK_4_-induced NO release, post-inflammatory cytokines (IL-6, TNF-α and MCP-1) secretions and intracellular NO generation in raw 264.7 cells. Biologically, SPE suppressed Pam_3_CSK_4_-induced expressions of cyclooxygenase-2 (COX-2), inducible nitric oxide synthase (iNOS), phosphorylation of NF-κB/p65 and IκBα, but did not significantly show effect on the proteins involved in MAPKs signaling (p38, ERK and JNK). The results were further confirmed by NF-κB-luc reporter gene assay and p65 nuclear translocation assay.

**Conclusions:**

In conclusion, SPE ameliorated Pam_3_CSK_4_-induced inflammation in raw 264.7 cells through suppressing TLR 1/2-mediated NF-κB activation.

**Electronic supplementary material:**

The online version of this article (10.1186/s13020-018-0193-x) contains supplementary material, which is available to authorized users.

## Background

Inflammation is an innate (non-specific) immune response and plays an important role in the physiological defense in response to various trauma or infection to the body [1]. An appropriate inflammatory response is necessary for the organism’s healing potential and facilitates tissue repair. However, an excessive or prolonged response might cause continuously damage to the body and induce many chronic diseases, organ dysfunction or organ failure [2, 3]. Therefore, an effective means of modulating systemic inflammation is beneficial for patients with chronic inflammatory autoimmune diseases, such as rheumatoid arthritis and diabetic nephropathy.

In the past decades, numerous studies indicated that transcription factors NF-κB target genes were involved in the occurrence and progress of various inflammations [4–8]. Activation of NF-κB stimulated macrophage recruitment and maturation, as well as the further production of pro-inflammatory cytokines and chemokines, such as tumor necrosis factor (TNF)-α, interleukin (IL)-1β, IL-6, monocyte chemoattractant protein (MCP)-1, and so on [9, 10]. Subsequently, the secreted inflammatory mediators further accelerated the degree of inflammation and the development of diseases [11]. On the other hand, being a family of transmembrane receptors closely related to the innate immune response [12], toll-like receptors (TLRs) (TLR1–TLR10 for human TLRs) present different functions on regulating inflammatory signaling and mediators based on their capacity to recognize the host derived agonists mostly released from the damaged cells or tissues during the progression of the diseases [13–16]. In triacyl lipoprotein-induced inflammations, the activation of NF-κB signaling pathways and production of various pro-inflammatory cytokines through TLR1/TLR2 (a heterodimer of TLR1 and TLR2) activation have been investigated and reported [17–20]. Therefore, targeting TLR1/TLR2 heterodimer-induced inflammation might be the potential therapeutic approach for such inflammatory diseases.

*Siegesbeckia pubescens* Makino (SP) is one of the plant origins of the traditional Chinese herbal medicine of Siegesbeckiae Herba, which has been widely used for various inflammatory diseases in China from the Tang dynasty. Currently, the chemical analysis indicated that SP mainly contained diterpenoids [21], sesquiterpenoids [22], flavonoids [23], glycosides [24] and some other constituents [25]. Moreover, the SP extracts or derived components were investigated to present various pharmacological activities such as anti-inflammatory [22, 26, 27], anti-allergic [28], and anti-cancer effects [29, 30]. The anti-inflammatory activity of SP was demonstrated to be related to its suppression on lipopolysaccharide (LPS)-induced nitric oxide (NO) [26] and inflammatory mediators [31] productions via NF-κB inactivation [32]. However, in our preliminary studies, the 50% ethanol extract of SP has been observed to have better activity against Pam_3_CSK_4_-(a specific TLR1/TLR2 agonist) than LPS-induced NO production in RAW 264.7 macrophages. In this study, the potential mechanisms of SP on Pam_3_CSK_4_-induced inflammation were further investigated and reported.

## Methods

The Minimum Standards of Reporting Checklist contains details of the experimental design, and statistics, and resources used in this study (Additional file 1).

### Chemical and reagents

Rutin, kirenol and darutoside (the purities of all standards were higher than 98% by HPLC analysis) were purchased from Chengdu Pufei De Biotech Co., Ltd. (Chengdu, China). Hoechst 33342, 3-(4, 5-dimethylthiazol-2-yl)-2,5-diphenyltetrazolium bromide (MTT) and Griess reagent were purchased from Sigma Chemicals Ltd. (St. Louis, MO, USA). Milli-Q water was prepared using a Milli-Q system (Millipore, MA, USA).

Dulbecco’s modified eagle medium (DMEM) and fetal bovine serum (FBS) were purchased from Gibco (Carlsbad, CA, USA). Pam_3_CSK_4_ (*N*-palmitoyl-*S*-[2,3-bis(palmitoyloxy)-(2RS)-propyl]-[*R*]-cysteinyl-[*S*]-seryl-[*S*]-lysyl-[*S*]-lysyl-[*S*]-lysyl-[*S*]-lysine·3HCl) was purchased from InvivoGen (San Diego, CA, USA). Enzyme-linked immunosorbent assay kits for IL-6, TNF-α, and MCP-1 were obtained from Neobioscience (Shenzhen, China). TurboFect transfection reagent was purchased from Thermo Fisher Scientific (Waltham, MA, USA). Antibodies were purchased from Santa Cruz Biotechnology (CA, USA) or Cell Signaling Tech (Danvers, MA, USA).

### Preparation and characterization of SP extract (SPE)

The herbal material of SP was collected from Guiyang (Guizhou province, China) and authenticated by the corresponding author. The voucher specimens (No. SP-002) were deposited at the Institute of Chinese Medical Sciences, University of Macau, Macao, China.

The powdered SP (100 g) was extracted twice with 50% ethanol (1:10, w/v) for 1 h each under reflux. The combined extracts were filtered with filter paper after cooling and then concentrated under reduced pressure to remove the ethanol. The powdered SPE (yield: 27.3%) was obtained by lyophilizing the concentrated sample with a Virtis Freeze Dryer (The Virtis Company, New York, USA).

Quantification of rutin, kirenol and darutoside in SPE was performed using an Agilent HP1100 system (Hewlett Packard, Agilent, USA) coupled with an Elite Hypersil BDS C-18 analytical column (100 mm × 2.1 mm I.D., 3 μm) (Dalian, China) maintained at 25 °C. Elution was performed with a mobile phase of A (0.2% phosphoric acid in water) and B (0.2% phosphoric acid in ACN) under a gradient program by a linear increase from 10% B to 22% B in the first 30 min, and to 23% B in 10 min, then to 30% in 30 min. The flow rate was 0.35 mL/min, and the injection volume was 10 μL. The analytes were monitored at the UV wavelength of 215 nm. Prior to next injection, the column was washed with 100% B for 5 min and then equilibrated with the initial mobile phase for 10 min.

### Cell culture

RAW 264.7 cells were obtained from the American Type Culture Collection (ATCC, Manassa, VA, USA). The cells were maintained in DMEM supplemented with 10% heat-inactivated FBS at 37 °C in humidified 5% CO_2_ atmosphere. The Minimum Standards of Reporting Checklist (Additional file 1) contains details of the experimental design, and statistics, and resources used in this study.

### Cytotoxicity

The cytotoxicity of SPE on RAW 264.7 cells was detected using the MTT assay combined with lactose dehydrogenase (LDH) assay. In brief, the cells were seeded on a 96-well plate (1 × 10^4^ cells/well) and allowed to adhere overnight. The cells were pretreated with SPE (25–200 μg/mL) for 4 h followed by co-treatment in the presence or absence of Pam_3_CSK_4_ (200 ng/mL) for 24 h. The cell proliferation was determined using the MTT assay as previous described [33]. The release of LDH in medium was determined using the LDH Cytotoxicity Detection Kit (ThermoFisher Scientific Inc., USA) according to the manufacturer’s instructions.

### Nitric oxide (NO) production and inflammatory cytokines secretion assays

RAW 264.7 cells were seeded on a 24-well plate (1 × 10^5^ cells/well) and allowed to adhere overnight. The cells were pretreatment with SPE (50, 100 and 200 μg/mL) or CU-CPT22 (4 µM, positive control) for 4 h and then co-treated with addition of Pam_3_CSK_4_ (200 ng/mL) for another 12 h. NO production was determined by measuring the accumulated nitrite in the culture medium with Griess reagent [33]. Cytokines (TNF-α, IL-6, and MCP-1) secretion in the supernatants of cultured cells was quantified using the enzyme-linked immunosorbent assay kits (Neobioscience, Shenzhen, China) following the manufacturer’s instructions.

### Capture of intracellular NO generation

RAW 264.7 cells were cultured in glass bottom dish overnight and pretreated with SPE (50, 100 and 200 μg/mL) for 4 h. Subsequently, the cells were co-treated with Pam_3_CSK_4_ (200 ng/mL) for 12 h and then washed twice with ice-cold PBS. After incubated with 4-amino-5-methylamino-2′,7′-difluorofluorescein diacetate (DAF-FM, 5 μM in PBS) (ThermoFisher Scientific Inc., USA) for 30 min at room temperature, the cells were washed with PBS and then stained with 1 μg/mL Hoechst 33342 for 10 min. The images were obtained by a Leica TCS SP8 laser scanning confocal microscopy (Leica Microsystem, Wetzlar, Germany).

### Western blotting analysis

RAW 264.7 cells were treated as described above. The harvested cells were washed thrice with ice-cold PBS and then extracted with RIPA buffer (Beyotime Biotechnology, Shanghai, China) containing protease inhibitor cocktails (ThermoFisher Scientific Inc., USA). The proteins (50 µg for each sample) were separated with SDS-PAGE (8%) and then transferred onto a PVDF membrane. The membranes were blocked with non-fat milk (5% in TBS containing 0.05% Tween-20, w/v) and incubated overnight at 4 °C with antibodies against iNOS, COX-2, p-IκBα, IκBα p-p65, p65 or GAPDH (1:1000). The membranes were then incubated with the corresponding secondary antibody (1:1000) at room temperature for 1 h. The signals were detected using ECL western blotting substrate (ThermoFisher Scientific Inc., USA) and ChemiDoc™ XRS+ system with Image Lab™ software (Bio-Rad Laboratories, Hercules, CA, USA).

### Immunofluorescence analysis

RAW 264.7 cells were attached on confocal dish overnight. After pretreatment with SPE (200 μg/mL) for 2 h, the cells were co-treated with Pam_3_CSK_4_ (200 ng/mL) for another 4 h and then fixed with 4% Paraformaldehyde (PFA) for 10 min at room temperature. The cells were washed thrice with PBS, permeabilized with 0.05% Triton X-100 in PBS for 3 min, and followed by blocking with 3% bovine serum albumin (in PBS, w/v) for 1 h. Thereafter, the cells were incubated with antibody against p65 (1:100) overnight and reacted with Alexa Fluor 488-conjugated secondary antibody (1:1000) for 1 h. The nuclear of the cell was stained with Hoechst 33342. The images were captured with a Leica TCS SP8 laser scanning confocal microscopy.

### Luciferase reporter gene assay

RAW 264.7 cells were transiently transfected with NF-κB-luc DNA for 48 h and refreshed with completed DMEM. The transfected cells were seeded in 6 plates overnight and pretreated with SPE (200 μg/mL) or CU-CPT22 (4 µM) for 2 h before Pam_3_CSK_4_ (200 ng/mL) stimulation for another 4 h. Luciferase activity was determined using a Dual-Glo luciferase assay system kit (Promega, Madison, Wisconsin, USA) following the manufacturer’s instructions.

### Statistical analysis

Each experiment was performed in triplicate and was repeated for at least thrice. All results were presented as mean ± SD. Variance between two groups was evaluated by one-way ANOVA using the GraphPad Prism software (GraphPad Software, United States). The Newman–Keuls multiple comparison tests were performed for post hoc pairwise comparisons. *P *< 0.05 was considered statistically significant difference.

## Results

### Characterization of SP extract

**Fig. 1:**
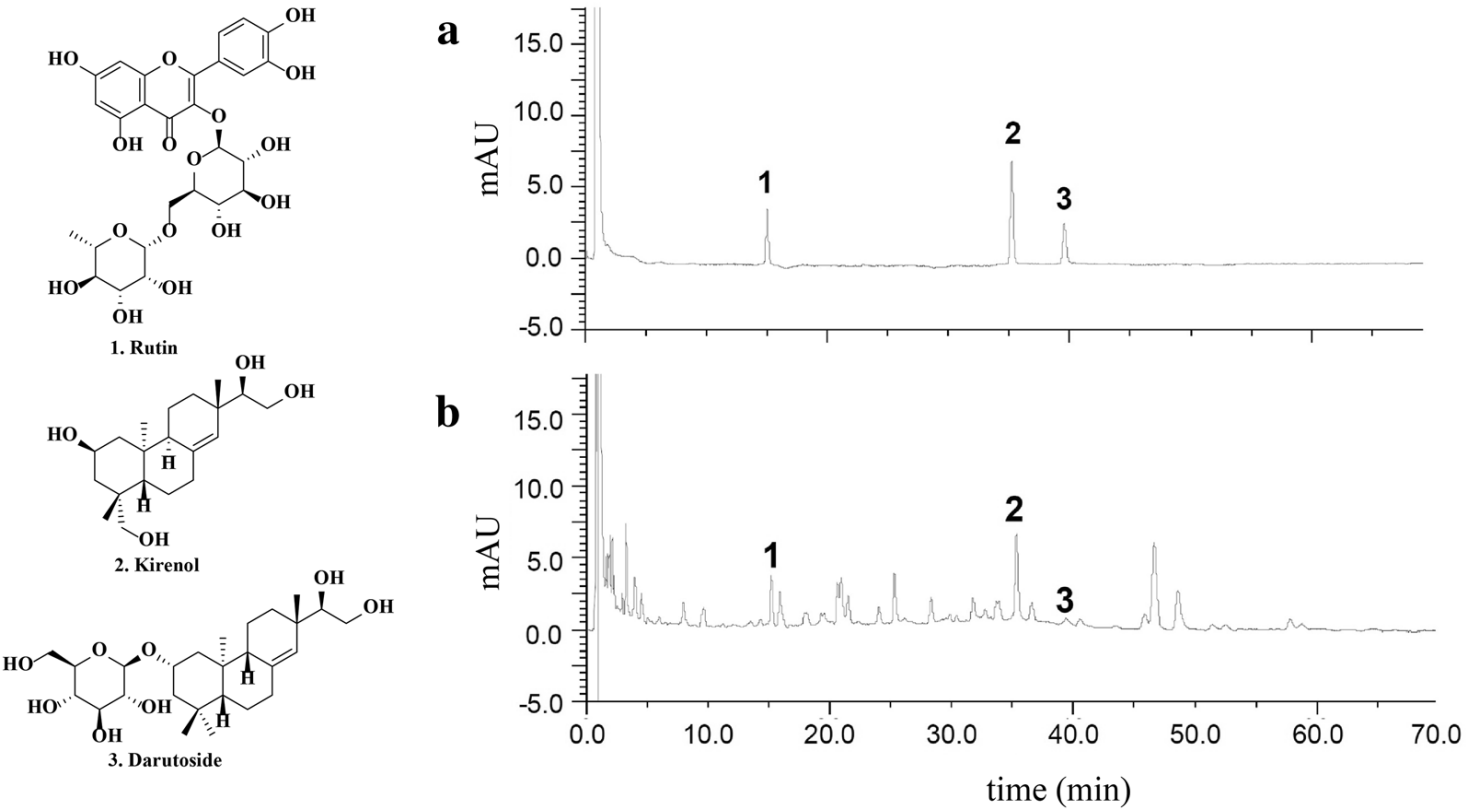
HPLC chromatograms of (**a**) mixed standards (7.5 μg/mL of rutin, kirenol and darutoside) and (**b**) SPE (1 mg/mL). **1**: rutin; **2**: kirenol; **3**: darutoside. The contents of rutin, kirenol and darutoside were determined to be 0.27 ± 0.01, 1.81 ± 0.02 and 0.28 ± 0.03%, respectively (*n *= 3)

The chromatograms of mixed standards and the SP extract were illustrated in Fig. 1. The contents of rutin, kirenol and darutoside in the extract were determined to be 0.27 ± 0.01, 1.81 ± 0.02 and 0.28 ± 0.03%, respectively.

### Cytotoxicity

**Fig. 2:**
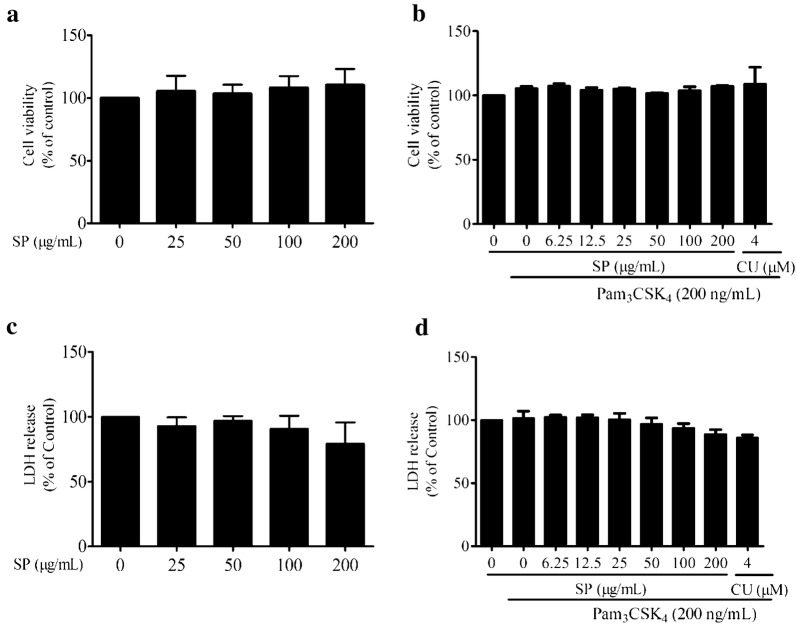
*Siegesbeckia pubescens* Makino (SP) or SP with Pam_3_CSK_4_-stimulated did not significantly affect the cell viability and cytotoxicity (*n *= 3). RAW 264.7 cells were treated with SP with different concentrations for 24 h. **a** The cell viability was measured by MTT assay and **c** cell cytotoxicity was measured by LDH assay. RAW 264.7 cells were pretreated with SP for 4 h before Pam_3_CSK_4_ (200 ng/mL) stimulation for another 12 h. **b** The cell viability was measured by MTT assay and **d** cell cytotoxicity was measured by LDH assay

The cytotoxicity of SPE on RAW 264.7 cells were determined using MTT and LDH assays. As illustrated in Fig. 2, SPE did not exert any observable toxicity on RAW 264.7 cells within the concentration ranged from 25 to 200 μg/mL while incubated with or without Pam_3_CSK_4_ (200 ng/mL) in 24 h. The concentrations of 50, 100 and 200 μg/mL were selected for SPE throughout the study.

### SPE suppressed NO release and inflammatory cytokines secretions in Pam_3_CSK_4_-induced RAW 264.7 cells

**Fig. 3:**
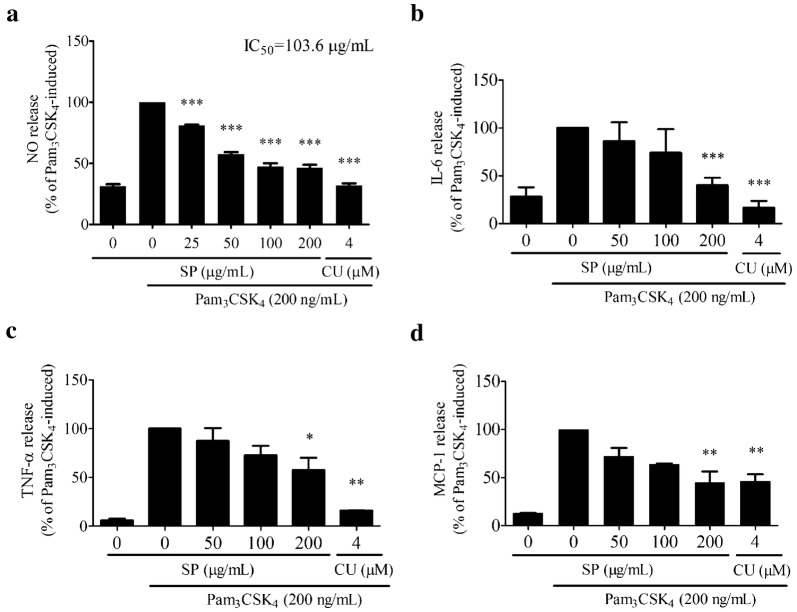
*Siegesbeckia pubescens* Makino (SP) exhibited anti-inflammatory effects in Pam_3_CSK_4_-stimulated RAW 264.7 cells (*n *= 3). RAW 264.7 cells were pretreated with SP with different concentrations for 4 h before Pam_3_CSK_4_ (200 ng/mL) stimulation for another 12 h. **a** Nitric oxide (NO) was determined by Griess assay. TLR1/TLR2 antagonist: CU-CPT22 (CU) was selected as the positive control. We measured the levels of **b** IL-6, **c** TNF-α, and **d** MCP-1 by ELISA assay. *P < 0.05 vs. Pam_3_CSK_4_-induced, **P < 0.01 vs. Pam_3_CSK_4_-induced, ***P < 0.001 vs. Pam_3_CSK_4_-induced

Compared to the normal control cells, incubation with Pam_3_CSK_4_ for 12 h significantly induced the release of NO and secretion of inflammatory cytokines (IL-6, TNF-α, and MCP-1) in RAW 264.7 cells (Fig. 3). However, the stimulations were dose-dependently inhibited by SPE in the concentration ranged from 50 to 200 μg/mL. More than 50% of Pam_3_CSK_4_-stimulated NO release was observed to be decreased (> 50%) by SPE at the concentration higher than 100 μg/mL (Fig. 3a). The estimated IC_50_ of SPE on NO release was calculated to be 103.6 µg/mL. Moreover, SPE (200 μg/mL) significantly inhibited the Pam_3_CSK_4_-induced IL-6 (59.98%), TNF-α (42.38%), and MCP-1 (55.10%) (Fig. 3b–d). The inhibitory effects of SPE on Pam_3_CSK_4_-induced inflammation were comparable to those of the positive control of CU-CPT22 (4 µM).

### SPE attenuated Pam_3_CSK_4_-induced intracellular NO generation

**Fig. 4:**
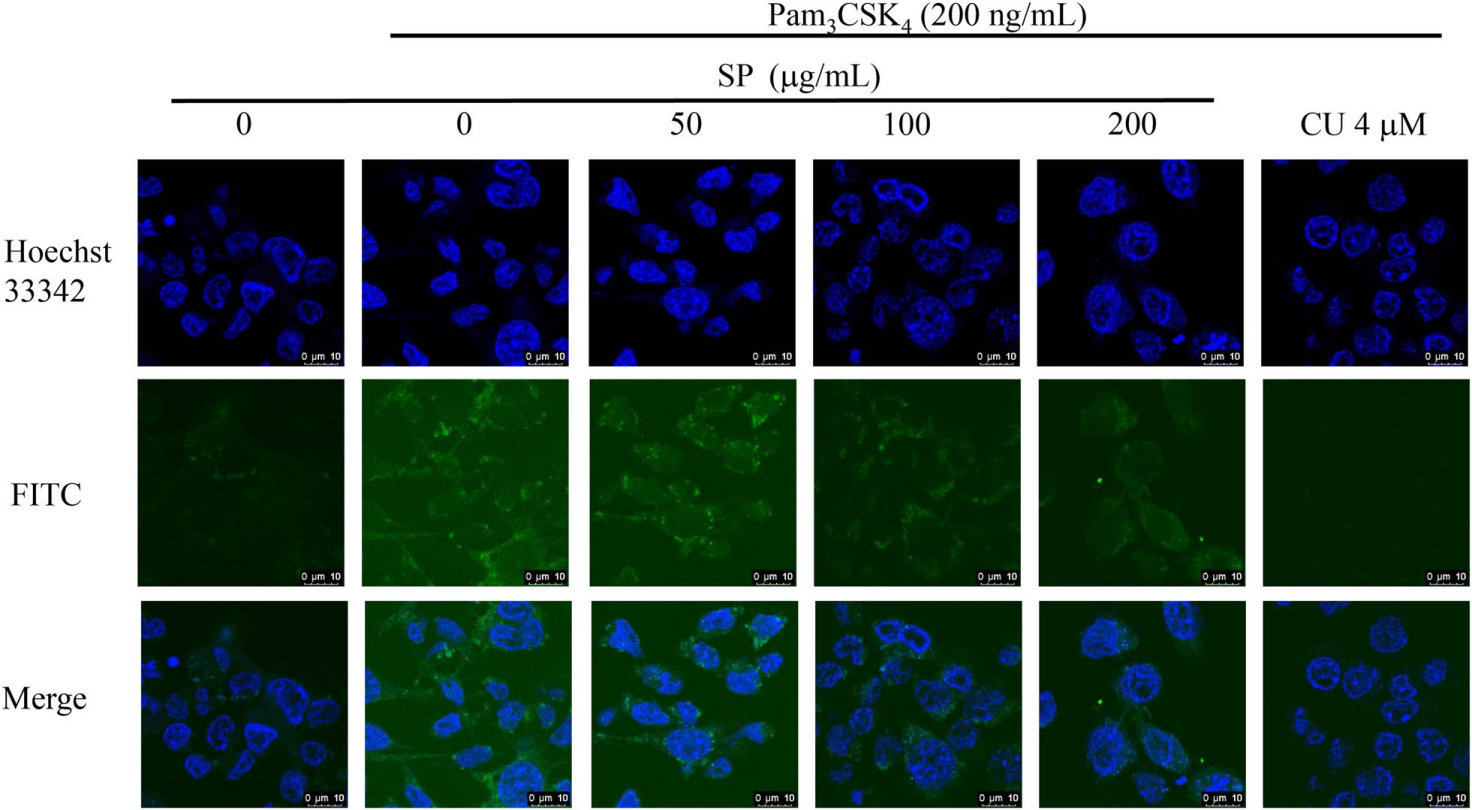
RAW 264.7 cells were pretreated with *Siegesbeckia pubescens* Makino (SP) (*n *= 3) for 4 h before Pam_3_CSK_4_ (200 ng/mL) stimulation for another 12 h. NO was captured by Leica TCS SP8 laser scanning confocal microscope with 5 μM DAF-FM diacetate (4-amino-5-methylamino-2′,7′-difluorofluorescein diacetate). CU-CPT22 (CU) is selected as the positive control

The inhibitory effect of SPE on Pam_3_CSK_4_-induced intracellular NO generation was determined by confocal microscopy. As illustrated in Fig. 4, Pam_3_CSK_4_ significantly stimulated the intracellular NO generation in RAW 264.7 cells. This stimulation was attenuated by SPE in a dose-dependent manner (50–200 μg/mL).

### SPE inhibited Pam_3_CSK_4_-induced the protein expressions of iNOS and COX-2

**Fig. 5:**
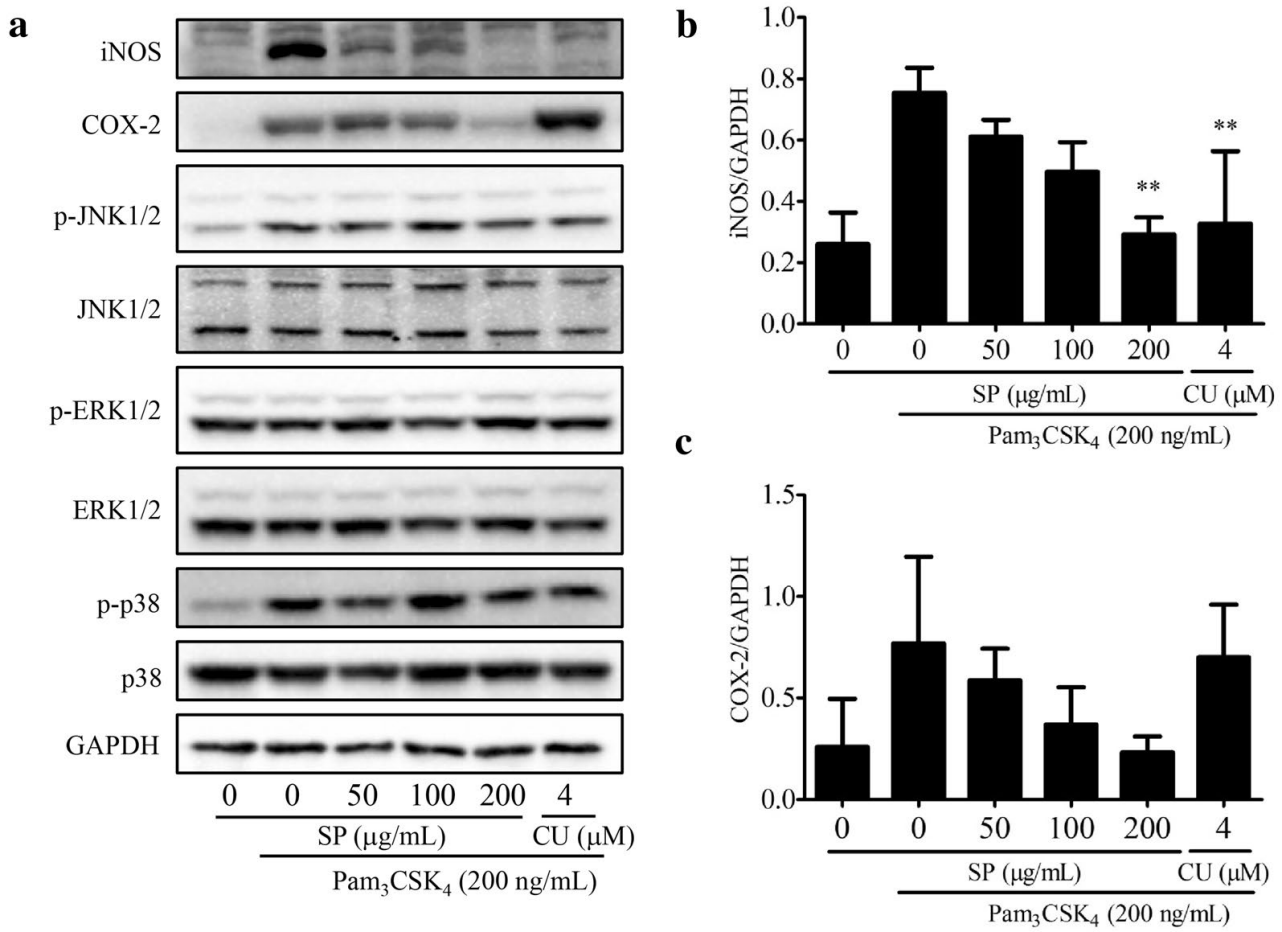
Effects of *Siegesbeckia pubescens* Makino (SP) on relevant pathways. RAW 264.7 cells were pretreated with SP (0, 50, 100 and 200 μg/mL) for 4 h and followed with Pam_3_CSK_4_ (200 ng/mL) addition for 12 h. CU-CPT22 (CU) is selected as the positive control. **a** Proteins was evaluated by Western blotting assay. **b**, **c** Quantification of iNOS and COX-2 protein was detected by densitometric analysis (*n *= 3). **P < 0.01 vs. Pam_3_CSK_4_-induced

The protein expressions of iNOS and COX-2 in SPE-treated RAW 264.7 cells were analyzed by Western blotting assay and illustrated in Fig. 5. SPE was determined to dose-dependently inhibit the Pam_3_CSK_4_-induced iNOS and COX-2 protein expressions in RAW 264.7 cells. The related amounts of iNOS and COX-2 in SPE-treated cells were determined to be decreased by 61.42 and 74.65% respectively when compared to those in the Pam_3_CSK_4_-induced cells (Fig. 5b, c). Moreover, the phosphorylation of JNK1/2 and p38, but not ERK1/2, was observed to be increased under Pam_3_CSK_4_ stimulation; whilst, SPE did not present any influence on the phosphorylation of such proteins.

### SPE inactivated Pam_3_CSK_4_-induced NF-κB signaling

**Fig. 6:**
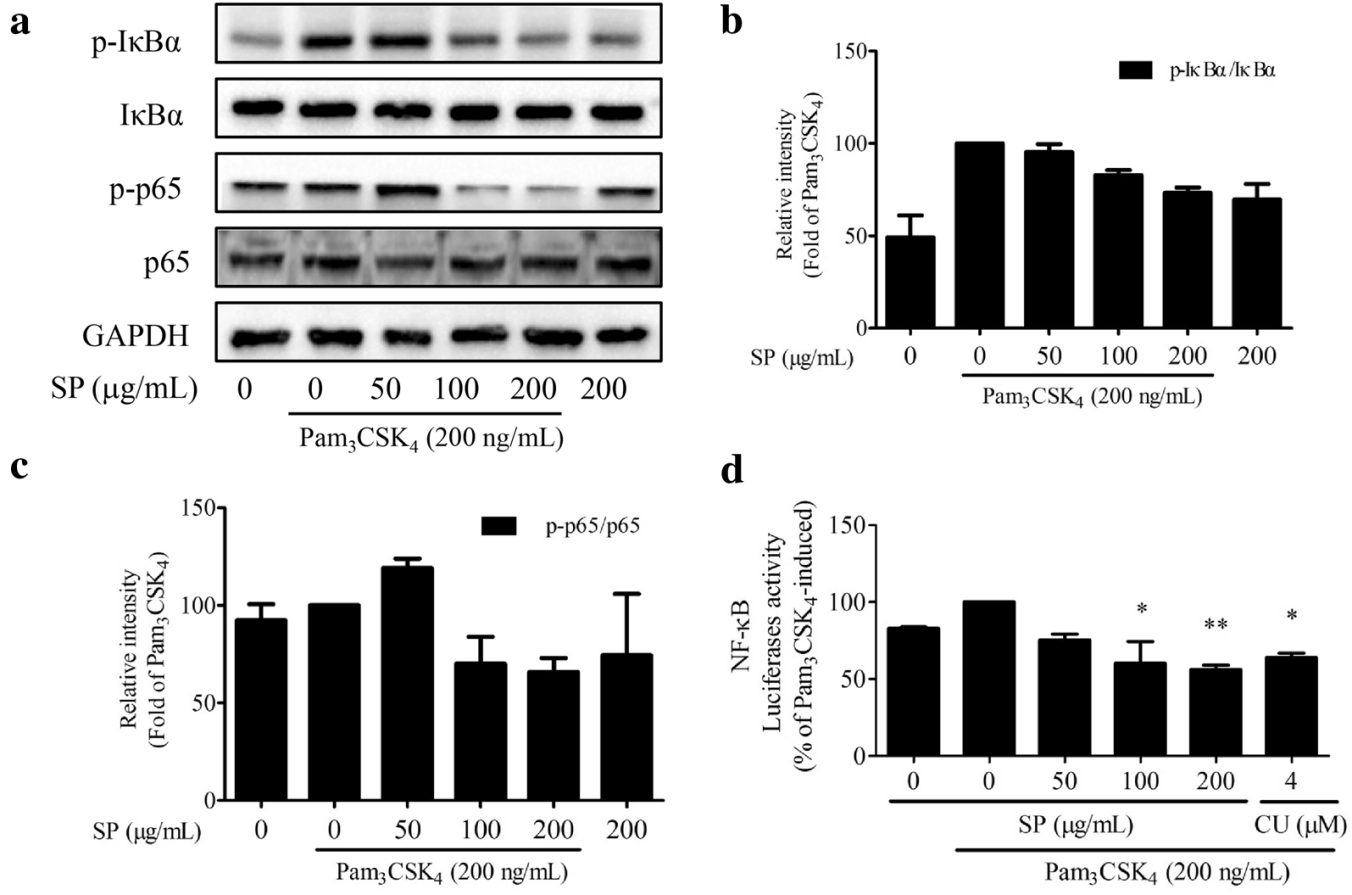
Effects of *Siegesbeckia pubescens* Makino (SP) on NF-κB pathways (*n *= 3). RAW 264.7 cells were pretreated with SP (0, 50, 100 and 200 μg/mL) for 2 h and followed with Pam_3_CSK_4_ (200 ng/mL) addition for 4 h. CU-CPT22 (CU) is selected as the positive control. **a** Proteins was evaluated by Western blotting assay. **b**, **c** Quantification was detected by densitometric analysis. **d** RAW 264.7 cells were transfected with NFκB-luc for 48 h. Cells were pretreated with SP 2 h before Pam_3_CSK_4_ (200 ng/mL) stimulation for another 4 h. Luciferase activity was determined by Dual-Glo Luciferase Assay. *P < 0.05 vs. Pam_3_CSK_4_-induced and **P < 0.01 vs. Pam_3_CSK_4_-induced

**Fig. 7:**
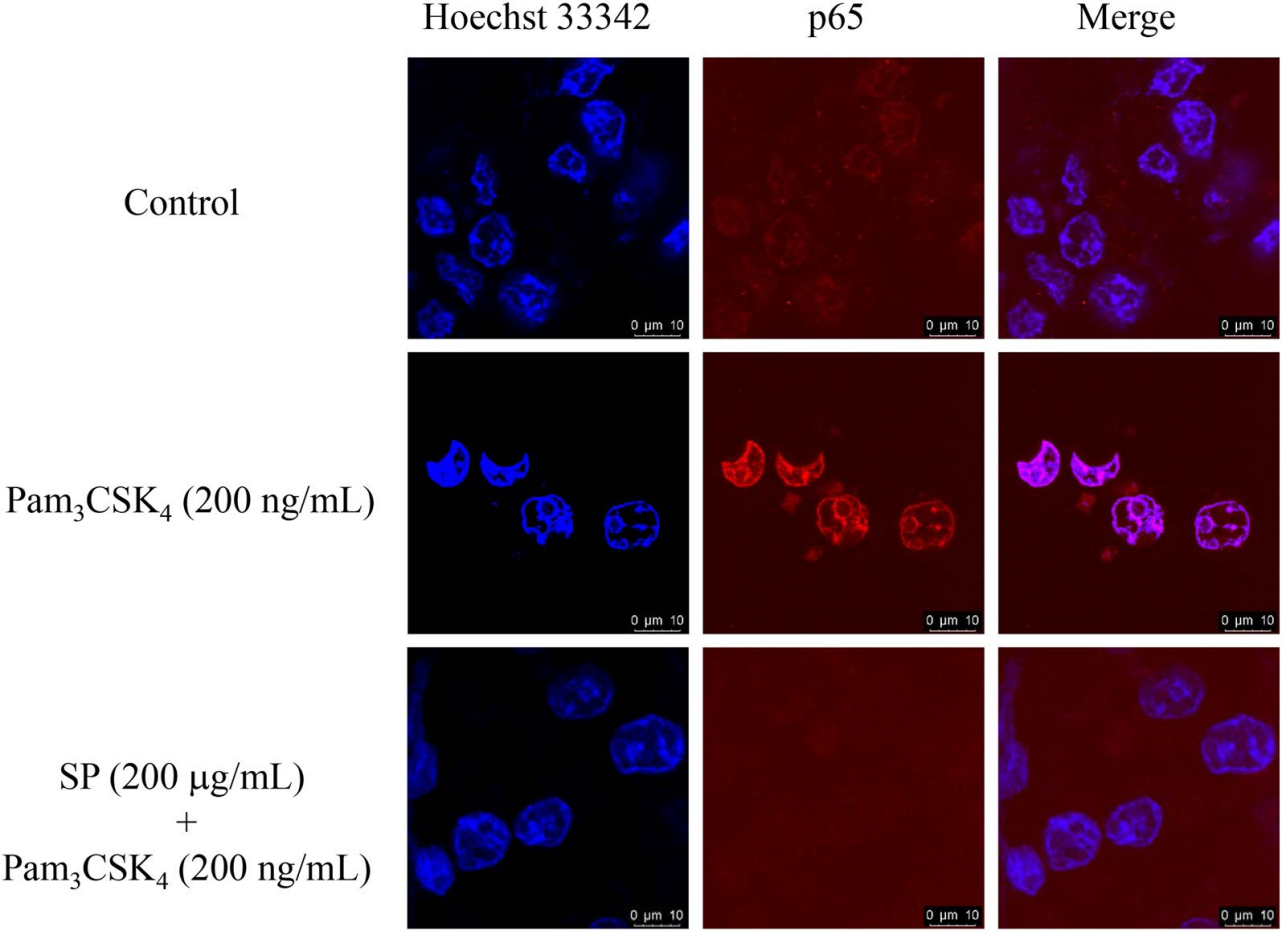
RAW 264.7 cells were pretreated with 200 μg/mL of *Siegesbeckia pubescens* Makino (SP) (*n *= 3) for 2 h before Pam_3_CSK_4_ stimulation for another 4 h. NF-κB/p65 nuclear translocation was determined by immunofluorescence assay

Compared to the untreated cell group, SPE at high concentration (200 μg/mL) was observed to slightly increase the phosphorylation of IκBα but did not influence on the phosphorylation of NF-κB-p65 (Fig. 6a–c). However, under the inflammatory condition, SPE presented dose-dependent inhibition on Pam_3_CSK_4_-induced phosphorylation of IκBα and NF-κB-p65 in RAW 264.7 cells. The activated p-IκBα and p-NF-κB-p65 were decreased by 26.71 and 34.14%, respectively, while co-treated with SPE at 200 μg/mL for 12 h (Fig. 6a–c). The results suggested the involvement of NF-κB inactivation in Pam_3_CSK_4_-induced inflammation by SPE. Confirmed by luciferase reporter gene assay, SP significantly attenuated the NF-κB-driven luciferase activity in Pam_3_CSK_4_-stimulated RAW 264.7 cells (Fig. 6d). Furthermore, the Pam_3_CSK_4_-induced p65 nuclear translocation was also determined to be attenuated by SPE using the immunofluorescence staining assay (Fig. 7).

## Discussion

In this work the anti-inflammatory effect and underlying mechanisms of SP on Pam_3_CSK_4_-induced inflammation in RAW 264.7 macrophages were investigated and reported.

TLRs have been demonstrated to play critical role in innate immune response in mammals against various infections. Up to date, 10 human TLRs (TLR1-TLR10) and 12 murine TLRs (TLR1-TLR9 and TLR11-TLR13) have been identified and demonstrated to react to various types of inflammations [13, 14]. Different to TLR4 (mainly response to LPS-induced inflammations), the TLR1–TLR2 heterodimer specifically responses to bacterial tri-acetylated lipopeptides or porins [34]. Activation of TLR1–TLR2 heterodimerization subsequently activates the NF-κB signaling [35] and MAPKs [17] pathways and up-regulates the inflammatory related proteins (such as iNOS and COX-2). Finally, the production of NO and secretion of inflammatory cytokines were increased. Using the specific TLR1–TLR2 heterodimerization stimulator of Pam_3_CSK_4_ [36], a synthetic tripalmitoylated lipopeptide with the similarity to the bacterial lipoproteins, the inflammatory responses are subsequently induced through activation on NF-κB signaling pathway.

Being a traditional anti-rheumatoid herbal medicine, SP was demonstrated to be beneficial to and has been applied for the management of various chronic inflammatory diseases [26–28]. The inactivation of TLR4-induced NF-κB signaling was identified to be involved in the biological mechanisms of SP on inhibiting the LPS-induced inflammations [32, 37]. However, our preliminary study, we observed that SPE presented more potency on suppressing Pam_3_CSK_4_-induced than LPS-induced NO release in RAW 264.7 macrophages. The results suggested the inhibition of SP on TLR1-TLR2 activation mediated inflammatory responses might be involved in its potential mechanisms on anti-inflammation. Further investigated in Pam_3_CSK_4_-stimulated macrophages, SPE improved the inflammatory responses of the cells by decreasing the NO release and cytokines (IL-6, TNF-α, and MCP-1) secretion into the culture medium. The biological mechanisms of such effect were identified to be associated with the suppressions of SPE on Pam_3_CSK_4_-stimulated NF-κB activation and up-regulation of the protein expressions of iNOS and COX-2. On the other hand, Pam_3_CSK_4_ stimulated inflammation by activating the MAPKs signaling, but SPE was determined to have no significant influences on the activated p38, ERK and JNK.

Previously, SP has been reported to contain multiple components. Diterpenoids, sesquiterpenoids and flavonoids have been determined to be the major components in SP [21–24]. Kirenol and darutoside, two *ent*-pimarane-type diterpenoids, were reported to be highly contained in SP [21, 38, 39]. Pharmacologically, the anti-inflammatory effects of SP were partially identified to be related to kirenol and darutoside [40]. Rutin, a widely distributed flavonoid in many plants, has been demonstrated to present various pharmacological activities such as anti-inflammation, anti-oxidant, anti-cancer as well as others [41]. In this study, the chemical compositions of SPE were analyzed using the HPLC method, the contents of the three representative components of kirenol (1.81 ± 0.02%), darutoside (0.28 ± 0.03%) and rutin (0.27 ± 0.01%) in SPE were quantified to be 2.36% in total. Further investigations on other chemical components in SPE as well as their relationship to TLR1-TLR2-activated inflammation are currently under progress in our research team.

## Conclusions

In conclusion, the anti-inflammatory activity of SP on Pam_3_CSK_4_-stimulated RAW 264.7 macrophages was investigated and reported. The results demonstrated that the 50% ethanol extract of SP could effectively decrease Pam_3_CSK_4_-induced NO release and cytokines secretion in RAW 264.7 cells. The potential biological mechanisms of SP on anti-inflammation were associated with its inactivation on Pam_3_CSK_4_-stimulated NF-κB signaling.

## Additional file

**Additional file 1.** Minimum Standards of Reporting Checklist.

## Abbreviations

SP: *Siegesbeckia pubescens* Makino
SPE: 50% ethanol extract of SP
NF-κB: nuclear factor-κB
MAPK: mitogen-activated protein kinase
TLR: toll-like receptor
Pam_3_CSK_4_: *N*-palmitoyl-*S*-[2,3-bis(palmitoyloxy)-(2*RS*)-propyl]-[*R*]-cysteinyl-[*S*]-seryl-[*S*]-lysyl-[*S*]-lysyl-[*S*]-lysyl-[*S*]-lysine·3HCl
NO: nitric oxide
COX-2: cyclooxygenase-2
iNOS: inducible nitric oxide synthase
IL-6: interleukin-6
IL-1β: interleukin-1β
TNF-α: tumor necrosis factor-α
MCP-1: monocyte chemoattractant protein-1
LPS: lipopolysaccharide
MTT: 3-(4,5-dimethylthiazol-2-yl)-2,5-diphenyltetrazolium bromide
DMEM: Dulbecco’s modified eagle medium
FBS: fetal bovine serum
LDH: lactose dehydrogenase
DAF-FM: 4-amino-5methylamino-2′,7′-difluorofluorescein diacetate
